# Telomerase activity of the Lugol-stained and -unstained squamous epithelia in the process of oesophageal carcinogenesis

**DOI:** 10.1054/bjoc.2001.2032

**Published:** 2001-09

**Authors:** M Inai, M Kano, Y Shimada, T Sakurai, T Chiba, M Imamura

**Affiliations:** Department of Gastroenterology and Hepatology, Graduate School of Medicine, Kyoto University, Kyoto, Japan; Department of Surgery and Surgical Basic Science, Kyoto University, Kyoto, Japan; Laboratory of Anatomic Pathology, Kyoto University Hospital, Kyoto University, Kyoto, Japan

**Keywords:** telomerase, Lugol, oesophagus, squamous cell carcinoma

## Abstract

Up-regulation of telomerase has been reported in many cancers. Our aim was to characterize telomerase activity in various states of the oesophagus to facilitate better understanding of carcinogenesis of oesophageal squamous cell carcinoma. During endoscopic examinations, we obtained 45 Lugol-stained normal epithelia, 31 Lugol-unstained epithelia (14 oesophagitis, 7 mild dysplasia, 5 severe dysplasia and 5 intramucosal cancer) and 9 advanced cancer. Telomerase activity was semi-quantified by a telomeric repeat amplification protocol using enzyme-linked immunosorbent assay, and expression of human telomerase reverse transcriptase mRNA was examined by in situ hybridization. In the Lugol-stained normal epithelia, telomerase activity increased in proportion to the increase of severity of the accompanying lesions, with a rank order of advanced cancer, intramucosal cancer, mild dysplasia and oesophagitis. In the Lugol-unstained lesions and advanced cancer, telomerase activity was highest in advanced cancer. Up-regulation of telomerase in normal squamous epithelium may be a marker of progression of oesophageal squamous cell carcinoma. Copyright 2001 Cancer Research Campaign © 2001 Cancer Research Campaignhttp://www.bjcancer.com


*British Journal of Cancer* (2001) **85**(7), 1006–1013
© 2001 Cancer Research Campaign
doi: 10.1054/ bjoc.2001.2032, available online at http://www.idealibrary.com on IDEAL

### Telomerase activity of the Lugol-stained and -unstained squamous epithelia in the process of oesophageal carcinogenesis

**M Inai[1,2], M Kano[2], Y Shimada[2], T Sakurai[3], T Chiba[1] and M Imamura[2]**

[1]Department of Gastroenterology and Hepatology, Graduate School of Medicine, Kyoto University, Kyoto, Japan; [2]Department of Surgery and Surgical Basic Science, Kyoto University, Kyoto, Japan; [3]Laboratory of Anatomic Pathology, Kyoto University Hospital, Kyoto University, Kyoto, Japan

**Summary** Up-regulation of telomerase has been reported in many cancers. Our aim was to characterize telomerase activity in various states of the oesophagus to facilitate better understanding of carcinogenesis of oesophageal squamous cell carcinoma. During endoscopic examinations, we obtained 45 Lugol-stained normal epithelia, 31 Lugol-unstained epithelia (14 oesophagitis, 7 mild dysplasia, 5 severe dysplasia and 5 intramucosal cancer) and 9 advanced cancer. Telomerase activity was semi-quantified by a telomeric repeat amplification protocol using enzyme-linked immunosorbent assay, and expression of human telomerase reverse transcriptase mRNA was examined by in situ hybridization. In the Lugol-stained normal epithelia, telomerase activity increased in proportion to the increase of severity of the accompanying lesions, with a rank order of advanced cancer, intramucosal cancer, mild dysplasia and oesophagitis. In the Lugol-unstained lesions and advanced cancer, telomerase activity was highest in advanced cancer. Up-regulation of telomerase in normal squamous epithelium may be a marker of progression of oesophageal squamous cell carcinoma. © 2001 Cancer Research Campaign http://www.bjcancer.com

**Keywords**: telomerase; Lugol; oesophagus; squamous cell carcinoma

Telomerase is a ribonucleoprotein that synthesizes telomeric sequence of genes and is important for the stability, replication and function of chromosomes (Morin, 1989). Numerous studies have demonstrated that telomerase activity is detected in 80–90% of human cancerous tissues as well as in most of the cancer cell lines (Chadeneau et al, 1995; Hiyama et al, 1995; Holt et al, 1996). In contrast, the activity has been shown to be absent in most normal human somatic tissues except the proliferative cells of the skin (Taylor et al, 1996) and those of the small intestine (Hiyama et al, 1996), and also normal T cells entering cell cycle (Yashima et al, 1997). Thus, telomerase is thought to be not only a new marker for malignancy but also a new target of anticancer therapy.

Oesophageal squamous cell carcinoma (ESC) is one of the most common cancers worldwide (Parkin et al, 1999). Its prognosis remains poor despite recent advances in both diagnosis and therapy. Indeed, the 5-year survival rate after surgery of patients with advanced ESC was 15.6%, and that of the patients with early ESC was 56.7% (Sugimachi et al, 1989). Moreover, multiple primary ESC is not uncommon (Kuwano et al, 1988; Pesko et al, 1994). These reports support the concept that the entire oesophagus may be considered as one entity of field carcinogenesis. The elucidation of the carcinogenesis of ESC may have a considerable effect on early diagnosis and therapy. In advanced ESC, similar to the other cancers, telomerase activity was detected in approximately 80% of the cases (Takubo et al, 1997), but telomerase activity was also present in a considerable number of normal oesophageal epithelia (Bachor et al, 1999; Ikeguchi et al, 1999). In order to elucidate the meaning of telomerase activity in normal oesophageal epithelium, we evaluated telomerase activity in both cancerous and non-cancerous epithelia of the oesophagus. For this purpose, we classified oesophageal epithelium according to the result of Lugol's iodine staining. Lugol's iodine staining is an available method to detect abnormal squamous mucosa easily, because mature squamous epithelium contains glycogen, which stains brown with iodine. When normal maturation is disturbed by inflammation, dysplasia or neoplasm, glycogen is decreased or absent, and the lesion remains unstained in contrast to the surrounding mucosa (Mori et al, 1993; Dawsey et al, 1998).

In addition, since human telomerase reverse transcriptase (hTERT), a component of human telomerase, plays a key role in activation of telomerase in cancer cells (Takakura et al, 1998; Kiaris and Schally, 1999), we did in situ hybridization for hTERT to clarify localization of telomerase activity in various oesophageal lesions and in Lugol-stained normal epithelia.

### MATERIALS AND METHODS

### Endoscopic sampling

All samples were obtained at Kyoto University hospital from July 1996 to April 1998. This study included 11 patients with advanced ESC and 35 patients who underwent routine endoscopic examination (46 in total). We obtained written informed consent for Lugol's staining and endoscopic sampling before endoscopic examination.

Each sample was obtained either by biopsy or by endoscopic mucosal resection. During endoscopic examination, the oesophagus was stained with 3% Lugol's solution and 2 samples were

*Received 12 December 2000*
*Revised 30 May 2001*
*Accepted 2 July 2001*

*Correspondence to:* Y Shimada

Maki Inai and Masato Kano equally contributed to this work.

obtained in the same Lugol-unstained lesion. 2 Lugol-stained epithelial samples were also obtained from each patient at least 3 cm distant from lesions. These 2 samples were taken close to each other. If no focal lesion was found, a standard site in the mid-oesophagus was sampled. One sample from each site was subjected to pathological examination and fixed in 10% buffered formalin embedded in paraffin, cut in 5 μm sections, and stained with H&E. The other was immediately frozen and stored in liquied nitrogen for measurement of telomerase activity. If the Lugol-unstained lesion was too small to take 2 samples, only 1 sample was obtained for pathological examination.

### Histological analysis

The sample slides were evaluated by 2 pathologists at our institution without knowledge of patient's history or endoscopic view. The histologic categories were based on criteria of the Japanese Society for Esophageal Disease (Japanese Society for Esophageal Disease, 1992). Dysplasia and ESC were classified as follows: dysplasia; nuclear atypia, such as enlargement, pleomorphism and hyperchromasia was present in the lower third (mild), in the lower two thirds or less (moderate), or in the two thirds or more (severe) of the epithelium; intramucosal ESC: malignant squamous cells were localized within the mucosal layer; advanced ESC: malignant squamous cells invaded through the muscular layer of mucosa. In this study, intraepithelial ESC was included in the severe dysplasia group because Japanese pathologists often make a diagnosis of intraepithelia ESC for lesions that Western pathologists consider to be severe dysplasia (Ronald et al, 2000).

### Telomerase activity

The sample was homogenized using pipette with ice-cold lysis buffer as described (Kim et al, 1994). After being kept on ice for 30 min, the sample was centrifuged for 30 min at 4˚C at 10000 ***g***. The supernatant was collected and snap frozen in liquid nitrogen before being stored at –80˚C. The protein concentration was determined by BCA protein assay kit (Pierce, Rockford, IL).

At first, we did telomeric repeat amplification protocol (TRAP) assay (Kim et al, 1994) adding an internal telomerase assay standard (Wright et al, 1995) to exclude the presence of polymerase chain reaction (PCR) inhibitors in protein extract (0.5 μg). Positive control (KYSE 273, the human ESC cell line (Shimada et al, 1992), which expressed telomerase activity) and negative control (no protein) were included in each assay. We analysed these PCR products by electrophoresis on 15% polyacrylamide gel and visualized them with SYBR green I nucleic acid gel stain (FMC, BioProducts, Rockland, ME).

Then, telomerase activity for semi-quantified analysis was determined by an additional modified TRAP assay combined with enzyme-linked immunosorbent assay (EIA) (Cheng et al, 1999). Primer sequences were described by Krupp et al (1997); (Klapper et al, 1998). Briefly, 0.5 μg of protein extract was incubated at 24˚C for 15 min in 50 μl TRAP reaction buffer (20 mM Tris (pH 8.3), 1 mM EGTA, 63 mM KCl, 0.005% Tween 20, 0.1 mg $ml^{-1}$ bovine serum albumin, 50 μM dNTP, 1.5 mM $MgCl_2$), 1 μg of biotinylated-TS primer [5′-AATCCGTCGAGCAGAGTT], 1 μg of digoxigeninated CX′ primer [5′-GTG(CCCTTA)$_3$CCCTAA], and 2 units of Taq DNA polymerase (GIBCO BRL, Grand Island, NY) followed by 31 cycles of PCR (94˚C for 30 s, 55˚C for 30 s, and 72˚C for 90 s). Positive control and negative control were also included in each assay. PCR products were transferred into streptavidin-coated microtitre plates (Boehringer Mannheim, Mannheim, Germany) and incubated at 30˚C for 1 h with horse-radish peroxidase-conjugated anti-digoxigenin (DIG) antibody (1:50) (Boehringer Mannheim). After washing with PBS, wells were incubated with tetramethylbenzidine substrate solution (Sigma Chemical Co, St Louis, MO) for 10 min at room temperature and was added to HCl. The absorbance was measured at a wavelength of 450 nm. All samples, positive control, and negative control were measured in triplicate and the mean optical density value was applied for analysis.

### Synthesis of DIG-labelled RNA probes

We extracted total RNA from KYSE 273 by acid guanidinium thiocyanate-phenol-chloroform extraction method (Chomczynski and Sacchi, 1987). In order to synthesize RNA probes, after reverse transcription using the First Strand cDNA Synthesis kit (Pharmacia Biotech, Uppsala, Sweden), cDNA corresponding to 1 μg of RNA was used as a PCR template. After heating at 94˚C for 5 min, 30 cycles of PCR were carried out (94˚C for 30 s, 60˚C for 30 s and 72˚C for 30 s). We designed hTERT primers as follows: 5′-TTGGGGATGAAGCGGAGTC and 5′-GGCTCCAGGCA-CAACGAAC (Nakamura et al, 1997). The 443 bp PCR products were cloned into pCR II plasmid vector using the TA cloning system (Invitrogen, Carlsbad, CA). To obtain RNA polymerase promoters on both sides of the fragment, the inserted fragment was excised by EcoRI and cloned into pBluescript II KS(+) (Stratagene, La Jolla, CA). We confirmed the sequence by ALF autosequencer (Pharmacia Biotech). The sequence between M13 forward and reverse primer site of this plasmid was amplified by these M13 primers (30 cycles of PCR; 94˚C for 30 s, 55˚C for 30 s and 72˚C for 30 s). In vitro transcription and labelling with DIG were made by DIG RNA labelling kit (Boehringer Mannheim). Transcribed products were recognized by gel electrophoresis and the contents of DIG-labelled probes were quantified by DIG nucleic acid detection kit (Boehringer Mannheim).

### In situ hybridization

Deparaffinized 4-μm-thick sections were incubated with 5 μg $ml^{-1}$ proteinase K (Boehringer Mannheim) at 37˚C for 10 min. After postfixation in 4% paraformaldehyde for 10 min, sections were immersed in 0.2 N HCl for 10 min. The sections were immersed in 0.1 mol $l^{-01}$ triethanolamine (TEA)-HCl (pH 8.0) for 1 min and acetylated with 0.25% acetic acid in 0.1 M TEA for 10 min. After washing with PBS, slides were dehydrated in graded ethanol series and dried. Hybridization was performed in a moist chamber with a cover glass at 50˚C for 16 h. The hybridization buffer contained 50% formamide, 10% dextran sulfate, 1 × Denhalt's solution, 0.25% sodium dodesyl sulfate, 200 μg $ml^{-1}$ *E. coli* transfer RNA, 10 mM Tris-HCl (pH 7.6), 0.6M NaCl, 1 mM EDTA and 3 μg $ml^{-1}$ of DIG-labelled RNA probe. The slides were then immersed in 50˚C with 5 × standard saline citrate (SSC), incubated in 2 × SSC containing 50% formamide at 50˚C for 30 min and immersed in TNE buffer (10 mM Tris-HCl [pH 7.6], 500 mM NaCl, 1 mM EDTA) at 37˚C for 10 min. After removal of single-stranded RNA with RNase A (GIBCO BRL) in TNE buffer, slides were washed with 2 × SSC, 0.2 × SSC twice and 0.1 × SSC at 50˚C for 20 min each. Signal was detected using DIG nucleic acid detection kit; sections were washed with buffer 1 and incubated in 1.5%

*© 2001 Cancer Research Campaign* 

blocking reagent for 1 h. After washing in buffer 1, the sections were incubated with 250 times diluted alkaline phosphatase-conjugated goat polyclonal anti-DIG Fab fragment antibody for 30 min, followed by washing in buffer 1. The second antibody was detected by revealing reagents containing nitroblue tetrazolium and 5-bromo-4-chloro-3-indoyl phosphate at room temperature for 16 h. The sections were then immersed in TE buffer, washed in distilled water and mounted with Crystal Mount (Biomeda Corp, Foster City, CA).

### Statistical analysis

Statistical analysis was performed by the Mann–Whitney U test and Scheffe's multiple comparison. Differences were judged statistically significant if the $P$ value was $<0.05$.

### RESULTS

### Telomerase activity in tissue samples

Tissue samples were obtained from 46 patients. 9 patients had history of reflux oesophagitis, but none of them were involved with the active phase of inflammation at the point of endoscopic examination. We collected 46 cases of Lugol-stained epithelia, 31 cases of Lugol-unstained lesions (14 oesophagitis, 7 mild dysplasia, 5 severe dysplasia and 5 intramucosal ESC) and 9 advanced ESC tissues (Table 1). Each of the samples did not contain PCR inhibitors on the results of modified TRAP assay, we carried out TRAP-EIA for all samples.

At first, we evaluated telomerase activity limited to the Lugol-stained epithelia. We divided them into 5 groups based on their accompanying lesions: Group A (= control group): not accompanied with any oesophageal lesions; Group B: accompanied by oesophagitis; Group C: accompanied by mild dysplasia; Group D: accompanied by severe dysplasia or intramucosal ESC; Group E: accompanied by advanced ESC. In this part, we treated severe dysplasia and intramucosal ESC in the same group, that is, as early ESC group. One Lugol-stained epithelium (Group E) was excluded due to lack of enough protein concentration. Mean telomerase activity of the Lugol-stained epithelia increased with the abnormal progression of accompanying oesophageal lesions (Figure 1A). Although Group E did not exhibit significantly higher mean telomerase activity compared with Group D, mean telomerase activity of Group E was the highest of the 5 Lugol-stained epithelial groups. Lugol-stained epithelia of Group C and Group D exhibited significantly higher mean telomerase activity than Group A. Telomerase activities in Group B and Group C varied among samples. The mean telomerase activity of Group C was higher than that of Group B, but there was no significant difference between them. The mean telomerase activity of Group B was significantly lower than that of Group D and Group E, but there was no significant difference between Group A and Group B.

Figure 1B shows telomerase activities of various Lugol-unstained lesions and advanced ESC. Although there was no significant difference between severe dysplasia and other lesions, mean telomerase activity of the Lugol-unstained epithelia increased with the severity of the lesions. There was the tendency that almost all the telomerase activities of cancerous lesions showed higher optical density value above 1.200.

As compared Lugol-stained epithelium with accompanied Lugol-unstained lesion in each group, mean telomerase activity was significantly higher in Lugol-unstained lesion with exception of Group C (Figure 2). In addition, almost all the telomerase activities were up-regulated in each Lugol-unstained lesion compared with the Lugol-stained epithelium obtained from the same patient. 9 Lugol-stained epithelia with history of reflux oesophagitis exhibited relatively high telomerase activity compared with other Lugol-stained epithelia. 6 of these 9 cases could be obtained both Lugol-stained epithelium and Lugol-unstained lesion. As for these 6 cases, 2 oesophagitis and 2 early ESC exhibited up-regulation of telomerase activity compared with Lugol-stained epithelium, but in 2 cases of mild dysplasia telomerase activity was not up-regulated.

### Expression of hTERT mRNA in the oesophageal tissues hTERT signal was revealed in nucleus and cytoplasm. We defined intensity of cytoplasm of the surface epithelium as control and compared relative signal intensity of various cells. In situ hybridization for hTERT in Lugol-stained epithelium revealed no apparent signal (Figure 3A) with a few exceptions. Lugol-stained epithelium expressed hTERT signal was obtained from a patient with intramucosal ESC (Figure 3B). In this sample, hTERT signal was observed in basal and para-basal layers and there were no cancer cells. In the oesophagitis, lymphocytes and regenerative epithelial cells expressed weak signal (Figure 3C). hTERT signal of mild dysplasia was observed in proliferative cells that showed mild

**Table 1** Summary of sample

| Group of stained epithelium[a] | Number of stained epithelium | Sampling number of accompanied lesion | | | | |
|---|---|---|---|---|---|---|
| | | Oesophagitis | Mild dysplasia | Severe dysplasia | Intramucosal squamous cell carcinoma | Advanced squamous cell carcinoma |
| A | 6 | – | – | – | – | |
| B | 14 | 7 | – | – | – | – |
| C | 6 | 0 | 5 | – | – | – |
| D | 9 | 2 | 2 | 3 | 5 | – |
| E | 11 | 5 | 0 | 2 | 0 | 9 |
| Total | 46 | 14 | 7 | 5 | 5 | 9 |

[a]Group A: not accompanied with any Lugol-unstained lesions or advanced squamous cell carcinoma; Group B: accompanied by oesophagitis; Group C: accompanied by mild dysplasia; Group D: accompanied by severe dysplasia or intramucosal squamous cell carcinoma; Group E: accompanied by advanced squamous cell carcinoma.

*© 2001 Cancer Research Campaign*

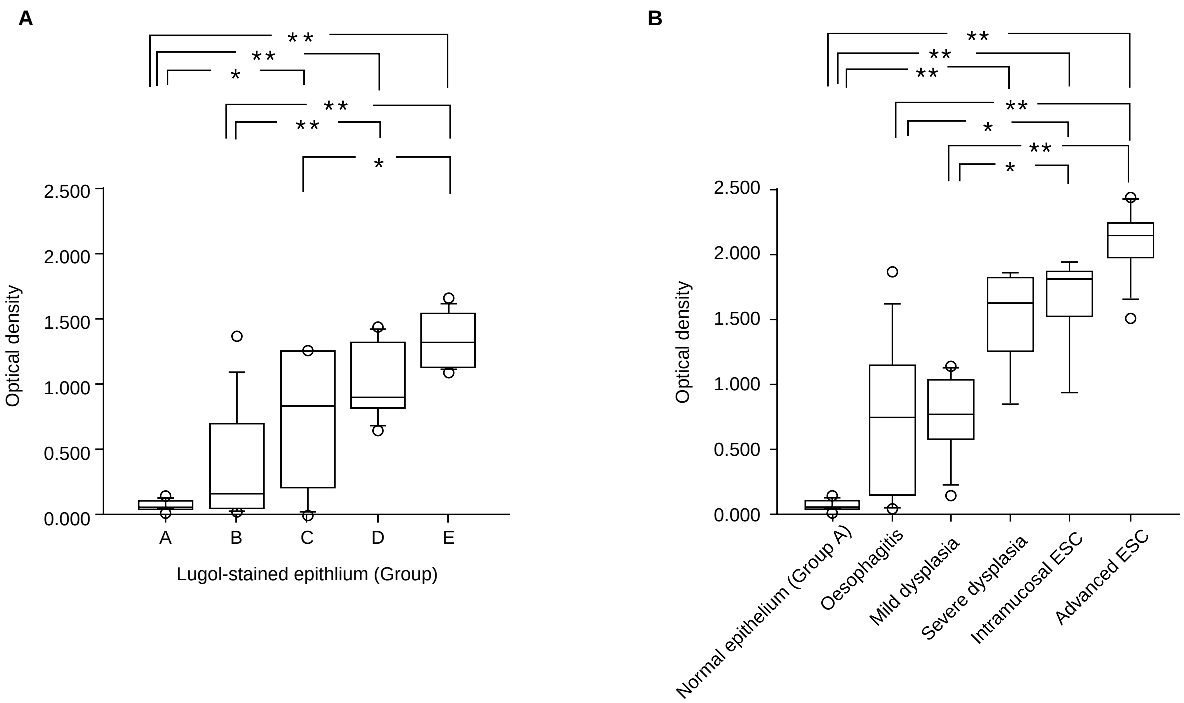

**Figure 1** Box-and-whisker plot of telomerase activity in samples. Telomerase activities were determined by TRAP-EIA. The bottom and top edges of the box mark the 25th and the 75th percentiles. The centre horizontal line is drawn at the sample median. The centre vertical lines drawn from the boxes extend to the 10th or 90th percentiles. Circles identify outside values. *$P < 0.05$; **$P < 0.01$. (**A**) Telomerase activity of the Lugol-stained epithelia classified by accompanying lesions. Group A: not accompanied with any lesions, Group B: accompanied by oesophagitis, Group C: accompanied by mild dysplasia, Group D: accompanied by early ESC (severe dysplasia or intramucosal ESC), Group E: accompanied by advanced ESC. (**B**) Telomerase activities of the Lugol-stained normal epithelia (Group A), Lugol-unstained epithelia (esophagitis, mild dysplasia, severe dysplasia, and intramucosal ESC), and advanced ESC

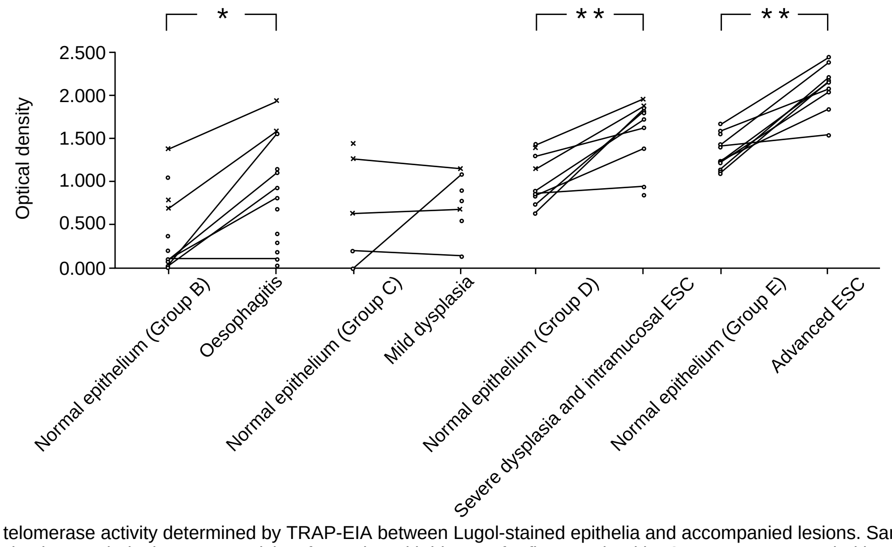

**Figure 2** Comparison of telomerase activity determined by TRAP-EIA between Lugol-stained epithelia and accompanied lesions. Samples obtained from same patient are connected. × is revealed telomerase activity of samples with history of reflux esophagitis. Group B: accompanied by esophagitis, Group C: accompanied by mild dysplasia, Group D: accompanied by early ESC (severe dysplasia or intramucosal ESC), Group E: accompanied by advanced ESC. *$P < 0.05$; **$P < 0.01$

atypia of nuclei (Figure 3D). Regenerative cells and lymphocytes and dysplastic cells showed a similar and relatively weak intensity. The signal intensity of basal and para-basal layer cells in Figure 3B was relatively higher than the above 2 samples.

On the other hand, almost all the advanced cancer cells clearly showed higher intensity than those described above (Figure 3F). The signal intensity of hTERT in the cytoplasm of cancer cells varied. Severe dysplasia and intramucosal ESC showed similar signal intensity with each other, and intramucosal ESC contained strong signal that was comparable to the signal of advanced ESC (Figure 3E).

There appeared to be a trend towards an increase in the intensity of hTERT signal with an increase in the severity of the cellular atypia in Lugol-unstained lesions and advanced ESC, but we could find no correlation between signal intensity of hTERT and telomerase activity in Lugol-stained epithelia and in oesophagitis (data

*© 2001 Cancer Research Campaign*

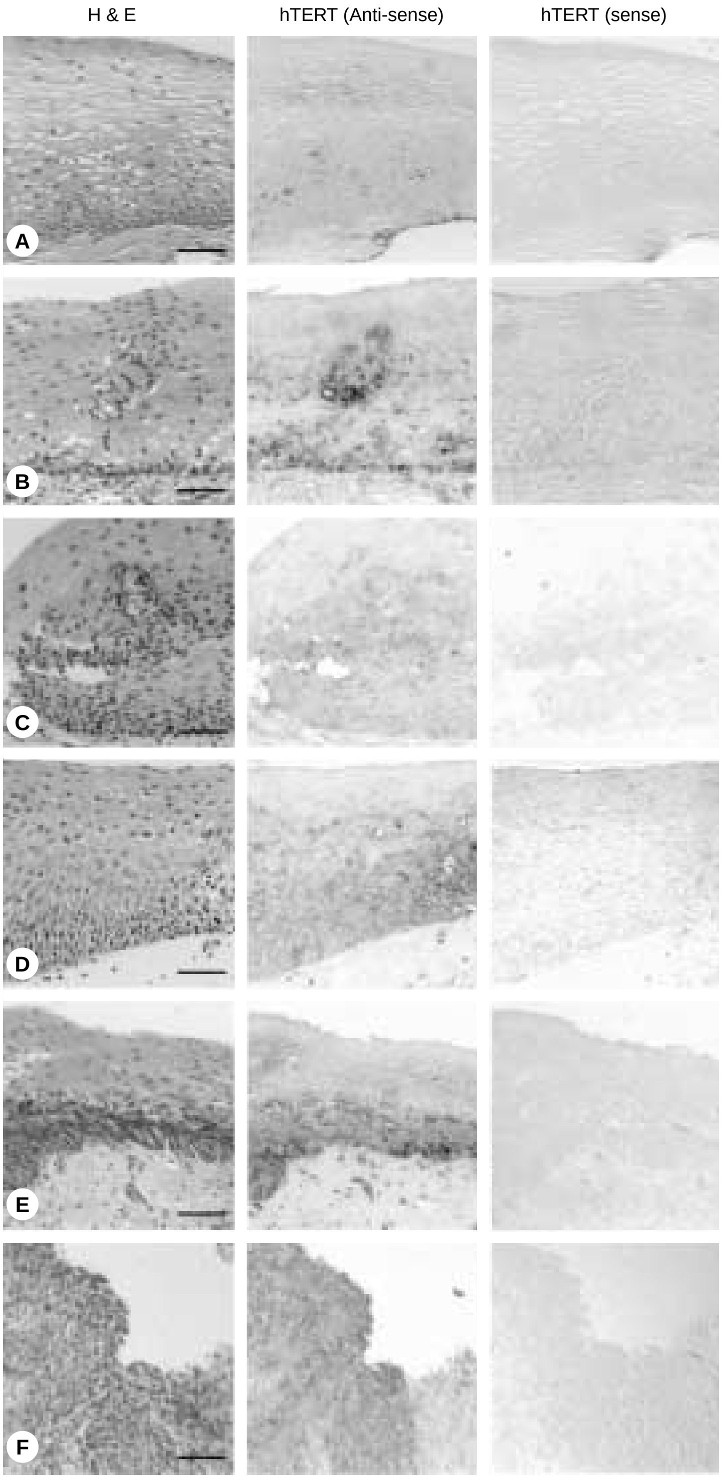

not shown).

**Figure 3** Distribution of hTERT mRNA signal in oesophageal tissues detected by in situ hybridization. No signal was present in Lugol-stained normal epithelia (**A**). hTERT signal was seen in basal and para-basal layers of Lugol-stained normal epithelia accompanied by intramucosal ESC (**B**). In oesophagitis, lymphocytes and regenerative cells exhibited weak signal (**C**). Intensity of dysplastic cells in mild dysplasia was similar to that of the regenerative cells of the esophagitis (**D**). Several cancer cells expressed strong signal in intramucosal ESC (**E**). Almost all the advanced cancer cells expressed strong intensity (**F**). Anti-sense; stained with anti-sense hTERT probe. Sense; stained with sense hTERT probe. Scale bars represent 50 μm

 *© 2001 Cancer Research Campaign*

### DISCUSSION

In this study, we semi-quantified telomerase activity in both cancerous and non-cancerous epithelia of the oesophagus using TRAP-EIA to elucidate the meaning of telomerase activity in various states of the oesophagus.

Interestingly, we found that the levels of telomerase activity in the Lugol-stained epithelia accompanied with ESC were very high and that telomerase activity of Lugol-stained epithelia increased with the progression of accompanying oesophageal lesions.

Because there are many reports that telomerase activity is absent in most normal human somatic tissues, it is unexpected that the Lugol-stained normal epithelia accompanying ESC exhibited such a high level of telomerase activity. Koyanagi et al reported that 10% of normal oesophageal tissue accompanied by advanced ESC showed telomerase activity which was derived from the microinvasion of cancer cells (Koyanagi et al, 1999). In our study, we detected no cancer cells in the Lugol-stained epithelia with H & E staining. Of course, it is difficult to completely rule out the presence of cancer cells in entire samples, but this is unlikely since almost all the telomerase activities in the Lugol-stained epithelia accompanying ESC were high in this study. This discrepancy may be ascribed to the difference of sampling method. They treated surgical specimens; on the other hand, we treated biopsy and EMR specimens Biopsy specimens can be frozen immediately compared with surgical specimens. Telomerase activity may decrease during the resection. Bachor et al also showed that biopsy samples in normal gastrointestinal mucosa exhibited high telomerase activity (Bachor et al, 1999). They explain that the difference between these 2 types of sampling may be caused by the difference of the proportion of connective tissue. It is known that connective tissue is telomerase-negative (Harle-Bachor and Boukamp, 1996) and may thereby dilute the activity of epithelial cells. In addition, it contains proteins that may inhibit Taq polymerase activity and prevent amplification of telomerase products (Nakamura et al, 1999).

We also found that some Lugol-stained epithelia in Group B or Group C showed high telomerase activities that was comparable to those in Group D or Group E. These epithelia in Group B and Group C were obtained from patients with a history of reflux oesophagitis with a few exceptions. The up-regulation of telomerase in Lugol-stained epithelia may be due to an acceleration of cell cycle to reproduce normal epithelial cell because telomerase is regarded as a biomarker of cell proliferation (Belair et al, 1997; Hytiroglou et al, 1998). Their endoscopic findings did not show the presence of active inflammation in the oesophagus but reproductive action may be continued after active phase of reflux oesophagitis. We did in situ hybridization for hTERT to clarify the derivation of telomerase activity. Although we could not detect hTERT signals in some Lugol-stained epithelia despite their high telomerase activity, Lugol-stained epithelia accompanied ESC expressed hTERT signals in basal and para-basal layers. Thus we assume that basal and para-basal cells will produce telomerase.

From these results, telomerase activity in Lugol-stained epithelium may show the cellular stability that is influenced by the process of carcinogenesis or reproduction. The oesophagus is easily affected by external stimulation, such as food intake, tobacco smoking, drinking, and so on, and then epithelial regeneration is repetitious in the entire oesophagus. This phenomenon is characteristic in the oesophagus and this may up-regulate telomerase activity in the normal oesophagus. Frequent regeneration and high telomerase activity may lead to canceration in the oesophagus. Indeed, the frequency of multiple ESC was higher in patients with abundant Lugol-unstained lesions than in patients with unicentric cancer and individuals who had no exposure to tobacco smoking and drinking would be less likely to exhibit small areas unstained (Nakanishi et al, 1998). A few studies also analysed telomerase activity in normal samples and showed the usefulness of telomerase activity to predict the clinical course of the diseases (Cheng et al, 1998).

According to the Lugol-unstained lesions and advanced ESC, telomerase activity increased relative to the severity of the disease. Similar results were reported concerning Lugol-unstained lesions, but there was no evaluation of oesophagitis and advanced ESC (Koyanagi et al, 2000). The wide range of telomerase activity in oesophagitis may show the degree of inflammation and the reproductive activity in each sample. Indeed, we demonstrated that lymphocytes and regenerative cells exhibited hTERT signal in the oesophagitis samples, though the signal was relatively weak. In this study, almost all the mild dysplasia exhibited higher telomerase activity than the Lugol-stained normal control Group A and mean telomerase activity of mild dysplasia was similar to that of oesophagitis. Moreover, the signal intensity of dysplastic cells was also relatively weak. This finding suggests that mild dysplasia would be an immature epithelium in the middle of the normal reproduction after inflammation or a neoplastic epithelium which grows very slowly. Severe dysplasia exhibited higher telomerase activity than mild dysplasia with no significant difference. This result would be due to the shortage of cases of severe dysplasia. Moreover, intramucosal ESC exhibited significantly higher mean telomerase activity than mild dysplasia. Additional up-regulation of telomerase was shown in advanced ESC. There was a similar tendency in the analysis of in situ hybridization. These data suggest that telomerase activity is up-regulated in the early stage of the oesophageal carcinogenesis and may be a critical role in the progression of ESC.

Figure 2 shows that telomerase activity of ESC increased compared with Lugol-stained epithelia. It seems that carcinogenesis would require not only telomerase activation but also some additional factors that could make further up-regulation of telomerase. The regulatory mechanism of telomerase activity is unknown at present. Several groups have demonstrated that c-*myc* mRNA or Myc participates in telomerase activation (Fujimoto and Takahashi, 1997; Wang et al, 1998), and that the TERT promoter contains c-Myc-binding sites that mediate TERT transcriptional activation (Wu et al, 1999). The amplification of c-*myc* is not frequent in the ESC (Lu et al, 1988; Esteve et al, 1993). On the other hand, overexpression of c-*myc* mRNA was detected in the normal mucosa adjacent to tumour in 23% of patients with ESC (Schrier and Peltenburg, 1993). One group has reported that the ectopic expression of the hTERT in combination with 2 oncogenes results in direct tumorigenic conversion of normal human epithelial cells (Hahn et al, 1999). Thus, the analysis of the regulatory mechanism of telomerase activity is important for the elucidation of carcinogenesis in the oesophagus.

In our study, there appeared to be a trend toward an increase of hTERT expression with an increase in the severity of the cellular atypia in Lugol-unstained lesions and advanced ESC, but there is no correlation between hTERT expression and telomerase activity in Lugol-stained epithelia and in oesophagitis. This disparity may be partially due to our own technical problem. But it also may be due to low abundance of this gene or short expression of hTERT

© 2001 Cancer Research Campaign 

mRNA in normal oesophageal samples compared with cancerous tissues. Indeed there are various reports about the relationship between telomerase activity level and the hTERT mRNA expression. In hepatic tissues, there was a clear correlation between telomerase activity level and the hTERT expression level (Nakayama et al, 1998). On the other hand, various levels of hTERT expression were observed in normal and cancer tissues without telomerase activity (Nakamura et al, 1999; Rohde et al, 2000). In an immunohistochemical study of the hTERT protein in colorectal tumour, telomerase activity and hTERT expression at both the mRNA and protein levels were found to be generally higher in the tumour part than in the non-tumour part, but expression of hTERT at mRNA or protein level did not always result in significant expression of telomerase activity (Tahara et al, 1999). Nakano et al were also unable to find a correlation between telomerase activity and hTERT expression and suggested that additional regulation, such as modulation of the hTERT gene product or coordination with other unknown proteins, may participate in the activation of telomerase (Nakano et al, 1998). To resolve these differences, we need some method which directly enables us to evaluate telomerase activity at the cellular level.

In conclusion, telomerase activity in Lugol-stained area may predict the development of oesophageal abnormal mucosa, especially, cancer. There is a possibility that high telomerase activity in normal oesophageal epithelium leads to a tendency to canceration and then the entire oesophagus may be considered as one entity of field carcinogenesis. Therefore, high telomerase activity in the Lugol-stained areas can not be used as a diagnostic marker for ESC but may be a marker of a high-risk group for ESC. Additional study is required to clarify the regulatory mechanism of telomerase activity in oesophageal carcinogenesis.

### REFERENCES

Bachor C, Bachor OA and Boukamp P (1999) Telomerase is active in normal gastrointestinal mucosa and not up-regulated in precancerous lesions. *J Cancer Res Clin Oncol* **125**: 453–460

Belair CD, Yeager TR, Lopez PM and Reznikoff CA (1997) Telomerase activity: a biomarker of cell proliferation, not malignant transformation. *Proc Natl Acad Sci USA* **94**: 13677–13682

Chadeneau C, Hay K, Hirte HW, Gallinger S and Bacchetti S (1995) Telomerase activity associated with acquisition of malignancy in human colorectal cancer. *Cancer Res* **55**: 2533–2536

Cheng AJ, Tang R, Wang JY, See LC and Wang TC (1998) Possible role of telomerase activation in the cancer predisposition of patients with hereditary nonpolyposis colorectal cancers. *J Natl Cancer Inst* **90**: 316–321

Cheng AJ, Tang R, Wang JY, Chang JT and Wang TC (1999) Polymerase Chain Reaction-based Enzyme Immunoassay for quantitation of telomerase activity: application to colorectal cancers. *Jpn J Cancer Res* **90**: 280–285

Chomczynski P and Sacchi N (1987) Single-step method of RNA isolation by acid guanidinium thiocyanate-phenol-chloroform extraction. *Anal Biochem* **162**: 156–159

Dawsey SM, Fleischer DE, Wang GQ, Zhou B, Kidwell JA, Lu N, Lewin KJ, Roth MJ, Tio TL and Taylor PR (1998) Mucosal iodine staining improves endoscopic visualization of squamous dysplasia and squamous cell carcinoma of the esophagus in Linxian, China. *Cancer* **83**: 220–231

Esteve A, Lehman T, Jiang W, Weinstein IB, Harris CC, Ruol A, Peracchia A, Montesano R and Hollstein M (1993) Correlation of p53 mutations with epidermal growth factor receptor overexpression and absence of mdm2 amplification in human esophageal carcinomas. *Mol Carcinog* **8**: 306–311

Fujimoto K and Takahashi M (1997) Telomerase activity in human leukemic cell lines is inhibited by antisense pentadecadeoxynucleotides targeted against c-myc mRNA. *Biochem Biophys Res Commun* **241**: 775–781

Hahn WC, Counter CM, Lundberg AS, Beijersbergen RL, Brooks MW and Weinberg RA (1999) Creation of human tumour cells with defined genetic elements. *Nature* **400**: 464–468

Harle-Bachor C and Boukamp P (1996) Telomerase activity in the regenerative basal layer of the epidermis in human skin and in immortal and carcinoma-derived skin keratinocytes. *Proc Natl Acad Sci U S A* **93**: 6476–6481

Hiyama E, Hiyama K, Tatsumoto N, Kodama T, Shay JW and Yokoyama T (1996) Telomerase activity in human intestine. *Int J Oncol* **9**: 453–458

Hiyama K, Hiyama E, Ishioka S, Yamakido M, Inai K, Gazdar AF, Piatyszek MA and Shay JW (1997) Telomerase activity in small-cell and non-small-cell lung cancers. *J Natl Cancer Inst* **87**: 895–902

Holt SE, Wright WE and Shay JW (1996) Regulation of telomerase activity in immortal cell lines. *Mol Cell Biol* **16**: 2932–2939

Hytiroglou P, Kotoula V, Thung SN, Tsokos M, Fiel MI and Papadimitriou CS (1998) Telomerase activity in precancerous hepatic nodules. *Cancer* **82**: 1831–1838

Ikeguchi M, Unate H, Maeta M and Kaibara N (1999) Detection of telomerase activity in esophageal squamous cell carcinoma and normal esophageal epithelium. *Langenbecks Arch Surg* **384**: 550–555

Japanese Society for Esophageal Disease (1992) Guidelines for the clinical and pathologic studies on carcinoma of the esophagus, 8th ed. Kanehara

Kiaris H and Schally AV (1999) Decrease in telomerase activity in U-87MG human glioblastomas after treatment with an antagonist of growth hormone-releasing hormone. *Proc Natl Acad Sci U S A* **96**: 226–231

Kim NW, Piatyszek MA, Prowse KR, Harley CB, West MD, Ho PL, Coviello GM, Wright WE, Weinrich SL and Shay JW (1994) Specific association of human telomerase activity with immortal cells and cancer. *Science* **266**: 2011–2015

Klapper W, Singh KK, Heidorn K, Parwaresch R and Krupp G (1998) Regulation of telomerase activity in quiescent immortalized human cells. *Biochim Biophys Acta* **1442**: 120–126

Koyanagi K, Ozawa S, Ando N, Takeuchi H, Ueda M and Kitajima M (1999) Clinical significance of telomerase activity in the non-cancerous epithelial region of oesophageal squamous cell carcinoma. *Br J Surg* **86**: 674–679

Koyanagi K, Ozawa S, Ando N, Mukai M, Kitagawa Y, Ueda M and Kitajima M (2000) Telomerase activity as an indicator of malignant potential in iodine-nonreactive lesions of the esophagus. *Cancer* **88**: 1524–1529

Krupp G, Kuhne K, Tamm S, Klapper W, Heidorn K, Rott A and Parwaresch R (1997) Molecular basis of artifacts in the detection of telomerase activity and a modified primer for a more robust 'TRAP' assay. *Nucleic Acids Res* **25**: 919–921

Kuwano H, Ohno S, Matsuda H, Mori M and Sugimachi K (1988) Serial histologic evaluation of multiple primary squamous cell carcinomas of the esophagus. *Cancer* **61**: 1635–1638

Lu SH, Hsieh LL, Luo FC and Weinstein IB (1988) Amplification of the EGF receptor and c-myc genes in human esophageal cancers. *Int J Cancer* **42**: 502–505

Mori M, Adachi Y, Matsushima T, Matsuda H, Kuwano H and Sugimachi K (1993) Lugol staining pattern and histology of esophageal lesions. *Am J Gastroenterol* **88**: 701–705

Morin GB (1989) The human telomere terminal transferase enzyme is a ribonucleoprotein that synthesizes TTAGGG repeats. *Cell* **59**: 521–529

Nakamura TM, Morin GB, Chapman KB, Weinrich SL, Andrews WH, Lingner J, Harley CB and Cech TR (1997) Telomerase catalytic subunit homologs from fission yeast and human. *Science* **277**: 955–959

Nakamura Y, Tahara E, Tahara H, Yasui W and Ide T (1999) Quantitative reevaluation of telomerase activity in cancerous and noncancerous gastrointestinal tissues. *Mol Carcinog* **26**: 312–320

Nakanishi Y, Ochiai A, Yoshimura K, Kato H, Shimoda T, Yamaguchi H, Tachimori Y, Watanabe H and Hirohashi S (1998) The clinicopathologic significance of small areas unstained by Lugol's iodine in the mucosa surrounding resected esophageal carcinoma. *Cancer* **82**: 1454–1459

Nakano K, Watney E and McDougall JK (1998) Telomerase activity and expression of telomerase RNA component and telomerase catalytic subunit gene in cervical cancer. *Am J Pathol* **153**: 857–864

Nakayama J, Tahara H, Tahara E, Saito M, Ito K, Nakamura H, Nakanishi T, Tahara E, Ide T and Ishikawa F (1998) Telomerase activation by hTRT in human normal fibroblasts and hepatocellular carcinomas. *Nat Genet* **18**: 65–68

Parkin DM, Pisani P and Ferlay J (1999) Estimates of the worldwide incidence of 25 major cancers in 1990. *Int J Cancer* **80**: 827–841

Rohde V, Sattler HP, Bund T, Bonkhoff H, Fixemer T, Bachmann C, Lensch R, Unteregger G, Stoeckle M and Wullich B (2000) Expression of the human telomerase reverse transcriptase is not related to telomerase activity in normal and malignant renal tissue. *Clin Cancer Res* **6**: 4803–4809

Schlemper RJ, Dawsey SM, Itabashi M, Iwashita A, Kato Y, Koike M, Lewin KJ, Riddell RH, Shimoda T, Sipponen P, Stolte M and Watanabe H (2000) Differences in diagnostic criteria for esophageal squamous cell carcinoma between Japanese and Western pathologists. *Cancer* **88**: 996–1006

Schrier PI and Peltenburg LT (1993) Relationship between myc oncogene activation and MHC class I expression. *Adv Cancer Res* **60**: 181–246

 *© 2001 Cancer Research Campaign*

Shimada Y, Imamura M, Wagata T, Yamaguchi N and Tobe T (1992) Characterization of 21 newly established esophageal cancer cell lines. *Cancer* **69**: 277–284

Sugimachi K, Ohno S, Matsuda H, Mori M, Matsuoka H and Kuwano H (1989) Clinicopathologic study of early stage esophageal carcinoma. *Surgery* **105**: 706–710

Tahara H, Yasui W, Tahara E, Fujimoto J, Ito K, Tamai K, Nakayama J, Ishikawa F, Tahara E and Ide T (1999) Immuno-histochemical detection of human telomerase catalytic component, hTERT, in human colorectal tumor and non-tumor tissue sections. *Oncogene* **18**: 1561–1567

Takakura M, Kyo S, Kanaya T, Tanaka M and Inoue M (1998) Expression of human telomerase subunits and correlation with telomerase activity in cervical cancer. *Cancer Res* **58**: 1558–1561

Takubo K, Nakamura K, Izumiyama N, Mafune K, Tanaka Y, Miyashita M, Sasajima K, Kato M and Oshimura M (1997) Telomerase activity in esophageal carcinoma. *J Surg Oncol* **66**: 88–92

Taylor RS, Ramirez RD, Ogoshi M, Chaffins M, Piatyszek MA and Shay JW (1996) Detection of telomerase activity in malignant and nonmalignant skin conditions. *J Invest Dermatol* **106**: 759–765

Wang J, Xie LY, Allan S, Beach D and Hannon GJ (1998) Myc activates telomerase. *Genes Dev* **12**: 1769–1774

Wright WE, Shay JW and Piatyszek MA (1995) Modifications of a telomeric repeat amplification protocol (TRAP) result in increased reliability, linearity and sensitivity. *Nucleic Acids Res* **23**: 3794–3795

Wu KJ, Grandori C, Amacker M, Simon-Vermot N, Polack A, Lingner J and Dalla-Favera R (1999) Direct activation of TERT transcription by c-MYC. *Nat Genet* **21**: 220–224

Yashima K, Piatyszek MA, Saboorian HM, Virmani AK, Brown D, Shay JW and Gazdar AF (1997) Telomerase activity and in situ telomerase RNA expression in malignant and non-malignant lymph nodes. *J Clin Pathol* **50**: 110–117

*© 2001 Cancer Research Campaign*

## References

[PDF_00607] Bachor C., Bachor O. A., Boukamp P. (1999). Telomerase is active in normal gastrointestinal mucosa and not up-regulated in precancerous lesions.. J Cancer Res Clin Oncol.

[PDF_00610] Belair C. D., Yeager T. R., Lopez P. M., Reznikoff C. A. (1997). Telomerase activity: a biomarker of cell proliferation, not malignant transformation.. Proc Natl Acad Sci U S A.

[PDF_00613] Chadeneau C., Hay K., Hirte H. W., Gallinger S., Bacchetti S. (1995). Telomerase activity associated with acquisition of malignancy in human colorectal cancer.. Cancer Res.

[PDF_00619] Cheng A. J., Tang R., Wang J. Y., Chang J. T., Wang T. C. (1999). Polymerase chain reaction-based enzyme immunoassay for quantitation of telomerase activity: application to colorectal cancers.. Jpn J Cancer Res.

[PDF_00616] Cheng A. J., Tang R., Wang J. Y., See L. C., Wang T. C. (1998). Possible role of telomerase activation in the cancer predisposition of patients with hereditary nonpolyposis colorectal cancers.. J Natl Cancer Inst.

[PDF_00622] Chomczynski P., Sacchi N. (1987). Single-step method of RNA isolation by acid guanidinium thiocyanate-phenol-chloroform extraction.. Anal Biochem.

[PDF_00625] Dawsey S. M., Fleischer D. E., Wang G. Q., Zhou B., Kidwell J. A., Lu N., Lewin K. J., Roth M. J., Tio T. L., Taylor P. R. (1998). Mucosal iodine staining improves endoscopic visualization of squamous dysplasia and squamous cell carcinoma of the esophagus in Linxian, China.. Cancer.

[PDF_00629] Esteve A., Lehman T., Jiang W., Weinstein I. B., Harris C. C., Ruol A., Peracchia A., Montesano R., Hollstein M. (1993). Correlation of p53 mutations with epidermal growth factor receptor overexpression and absence of mdm2 amplification in human esophageal carcinomas.. Mol Carcinog.

[PDF_00633] Fujimoto K., Takahashi M. (1997). Telomerase activity in human leukemic cell lines is inhibited by antisense pentadecadeoxynucleotides targeted against c-myc mRNA.. Biochem Biophys Res Commun.

[PDF_00636] Hahn W. C., Counter C. M., Lundberg A. S., Beijersbergen R. L., Brooks M. W., Weinberg R. A. (1999). Creation of human tumour cells with defined genetic elements.. Nature.

[PDF_00644] Hiyama K., Hiyama E., Ishioka S., Yamakido M., Inai K., Gazdar A. F., Piatyszek M. A., Shay J. W. (1995). Telomerase activity in small-cell and non-small-cell lung cancers.. J Natl Cancer Inst.

[PDF_00647] Holt S. E., Wright W. E., Shay J. W. (1996). Regulation of telomerase activity in immortal cell lines.. Mol Cell Biol.

[PDF_00649] Hytiroglou P., Kotoula V., Thung S. N., Tsokos M., Fiel M. I., Papadimitriou C. S. (1998). Telomerase activity in precancerous hepatic nodules.. Cancer.

[PDF_00639] Härle-Bachor C., Boukamp P. (1996). Telomerase activity in the regenerative basal layer of the epidermis inhuman skin and in immortal and carcinoma-derived skin keratinocytes.. Proc Natl Acad Sci U S A.

[PDF_00652] Ikeguchi M., Unate H., Maeta M., Kaibara N. (1999). Detection of telomerase activity in esophageal squamous cell carcinoma and normal esophageal epithelium.. Langenbecks Arch Surg.

[PDF_00657] Kiaris H., Schally A. V. (1999). Decrease in telomerase activity in U-87MG human glioblastomas after treatment with an antagonist of growth hormone-releasing hormone.. Proc Natl Acad Sci U S A.

[PDF_00660] Kim N. W., Piatyszek M. A., Prowse K. R., Harley C. B., West M. D., Ho P. L., Coviello G. M., Wright W. E., Weinrich S. L., Shay J. W. (1994). Specific association of human telomerase activity with immortal cells and cancer.. Science.

[PDF_00663] Klapper W., Singh K. K., Heidorn K., Parwaresch R., Krupp G. (1998). Regulation of telomerase activity in quiescent immortalized human cells.. Biochim Biophys Acta.

[PDF_00669] Koyanagi K., Ozawa S., Ando N., Mukai M., Kitagawa Y., Ueda M., Kitajima M. (2000). Telomerase activity as an indicator of malignant potential in iodine-nonreactive lesions of the esophagus.. Cancer.

[PDF_00666] Koyanagi K., Ozawa S., Ando N., Takeuchi H., Ueda M., Kitajima M. (1999). Clinical significance of telomerase activity in the non-cancerous epithelial region of oesophageal squamous cell carcinoma.. Br J Surg.

[PDF_00672] Krupp G., Kühne K., Tamm S., Klapper W., Heidorn K., Rott A., Parwaresch R. (1997). Molecular basis of artifacts in the detection of telomerase activity and a modified primer for a more robust 'TRAP' assay.. Nucleic Acids Res.

[PDF_00675] Kuwano H., Ohno S., Matsuda H., Mori M., Sugimachi K. (1988). Serial histologic evaluation of multiple primary squamous cell carcinomas of the esophagus.. Cancer.

[PDF_00678] Lu S. H., Hsieh L. L., Luo F. C., Weinstein I. B. (1988). Amplification of the EGF receptor and c-myc genes in human esophageal cancers.. Int J Cancer.

[PDF_00680] Mori M., Adachi Y., Matsushima T., Matsuda H., Kuwano H., Sugimachi K. (1993). Lugol staining pattern and histology of esophageal lesions.. Am J Gastroenterol.

[PDF_00683] Morin G. B. (1989). The human telomere terminal transferase enzyme is a ribonucleoprotein that synthesizes TTAGGG repeats.. Cell.

[PDF_00685] Nakamura T. M., Morin G. B., Chapman K. B., Weinrich S. L., Andrews W. H., Lingner J., Harley C. B., Cech T. R. (1997). Telomerase catalytic subunit homologs from fission yeast and human.. Science.

[PDF_00688] Nakamura Y., Tahara E., Tahara H., Yasui W., Ide T. (1999). Quantitative reevaluation of telomerase activity in cancerous and noncancerous gastrointestinal tissues.. Mol Carcinog.

[PDF_00691] Nakanishi Y., Ochiai A., Yoshimura K., Kato H., Shimoda T., Yamaguchi H., Tachimori Y., Watanabe H., Hirohashi S. (1998). The clinicopathologic significance of small areas unstained by Lugol's iodine in the mucosa surrounding resected esophageal carcinoma: an analysis of 147 cases.. Cancer.

[PDF_00695] Nakano K., Watney E., McDougall J. K. (1998). Telomerase activity and expression of telomerase RNA component and telomerase catalytic subunit gene in cervical cancer.. Am J Pathol.

[PDF_00698] Nakayama J., Tahara H., Tahara E., Saito M., Ito K., Nakamura H., Nakanishi T., Tahara E., Ide T., Ishikawa F. (1998). Telomerase activation by hTRT in human normal fibroblasts and hepatocellular carcinomas.. Nat Genet.

[PDF_00701] Parkin D. M., Pisani P., Ferlay J. (1999). Estimates of the worldwide incidence of 25 major cancers in 1990.. Int J Cancer.

[PDF_00703] Rohde V., Sattler H. P., Bund T., Bonkhoff H., Fixemer T., Bachmann C., Lensch R., Unteregger G., Stoeckle M., Wullich B. (2000). Expression of the human telomerase reverse transcriptase is not related to telomerase activity in normal and malignant renal tissue.. Clin Cancer Res.

[PDF_00707] Schlemper R. J., Dawsey S. M., Itabashi M., Iwashita A., Kato Y., Koike M., Lewin K. J., Riddell R. H., Shimoda T., Sipponen P. (2000). Differences in diagnostic criteria for esophageal squamous cell carcinoma between Japanese and Western pathologists.. Cancer.

[PDF_00711] Schrier P. I., Peltenburg L. T. (1993). Relationship between myc oncogene activation and MHC class I expression.. Adv Cancer Res.

[PDF_00716] Shimada Y., Imamura M., Wagata T., Yamaguchi N., Tobe T. (1992). Characterization of 21 newly established esophageal cancer cell lines.. Cancer.

[PDF_00719] Sugimachi K., Ohno S., Matsuda H., Mori M., Matsuoka H., Kuwano H. (1989). Clinicopathologic study of early stage esophageal carcinoma.. Surgery.

[PDF_00722] Tahara H., Yasui W., Tahara E., Fujimoto J., Ito K., Tamai K., Nakayama J., Ishikawa F., Tahara E., Ide T. (1999). Immuno-histochemical detection of human telomerase catalytic component, hTERT, in human colorectal tumor and non-tumor tissue sections.. Oncogene.

[PDF_00726] Takakura M., Kyo S., Kanaya T., Tanaka M., Inoue M. (1998). Expression of human telomerase subunits and correlation with telomerase activity in cervical cancer.. Cancer Res.

[PDF_00729] Takubo K., Nakamura K., Izumiyama N., Mafune K., Tanaka Y., Miyashita M., Sasajima K., Kato M., Oshimura M. (1997). Telomerase activity in esophageal carcinoma.. J Surg Oncol.

[PDF_00732] Taylor R. S., Ramirez R. D., Ogoshi M., Chaffins M., Piatyszek M. A., Shay J. W. (1996). Detection of telomerase activity in malignant and nonmalignant skin conditions.. J Invest Dermatol.

[PDF_00735] Wang J., Xie L. Y., Allan S., Beach D., Hannon G. J. (1998). Myc activates telomerase.. Genes Dev.

[PDF_00737] Wright W. E., Shay J. W., Piatyszek M. A. (1995). Modifications of a telomeric repeat amplification protocol (TRAP) result in increased reliability, linearity and sensitivity.. Nucleic Acids Res.

[PDF_00741] Wu K. J., Grandori C., Amacker M., Simon-Vermot N., Polack A., Lingner J., Dalla-Favera R. (1999). Direct activation of TERT transcription by c-MYC.. Nat Genet.

[PDF_00743] Yashima K., Piatyszek M. A., Saboorian H. M., Virmani A. K., Brown D., Shay J. W., Gazdar A. F. (1997). Telomerase activity and in situ telomerase RNA expression in malignant and non-malignant lymph nodes.. J Clin Pathol.

